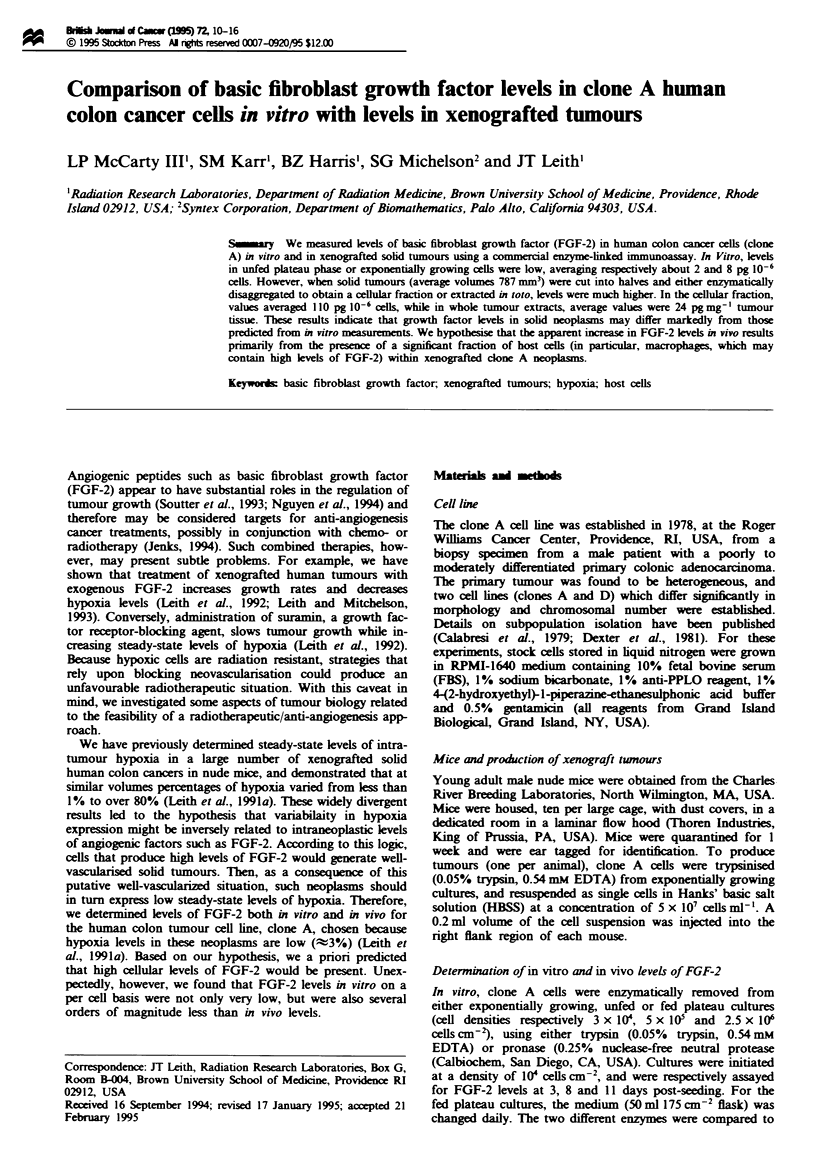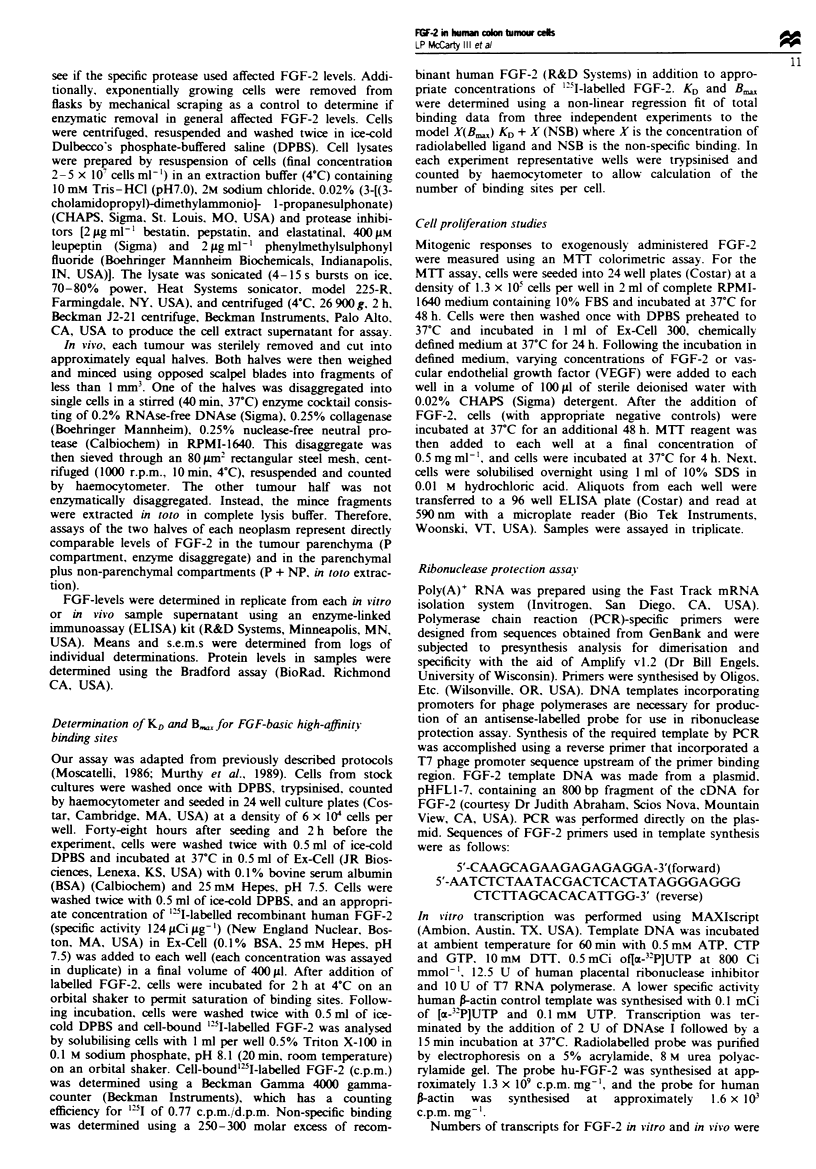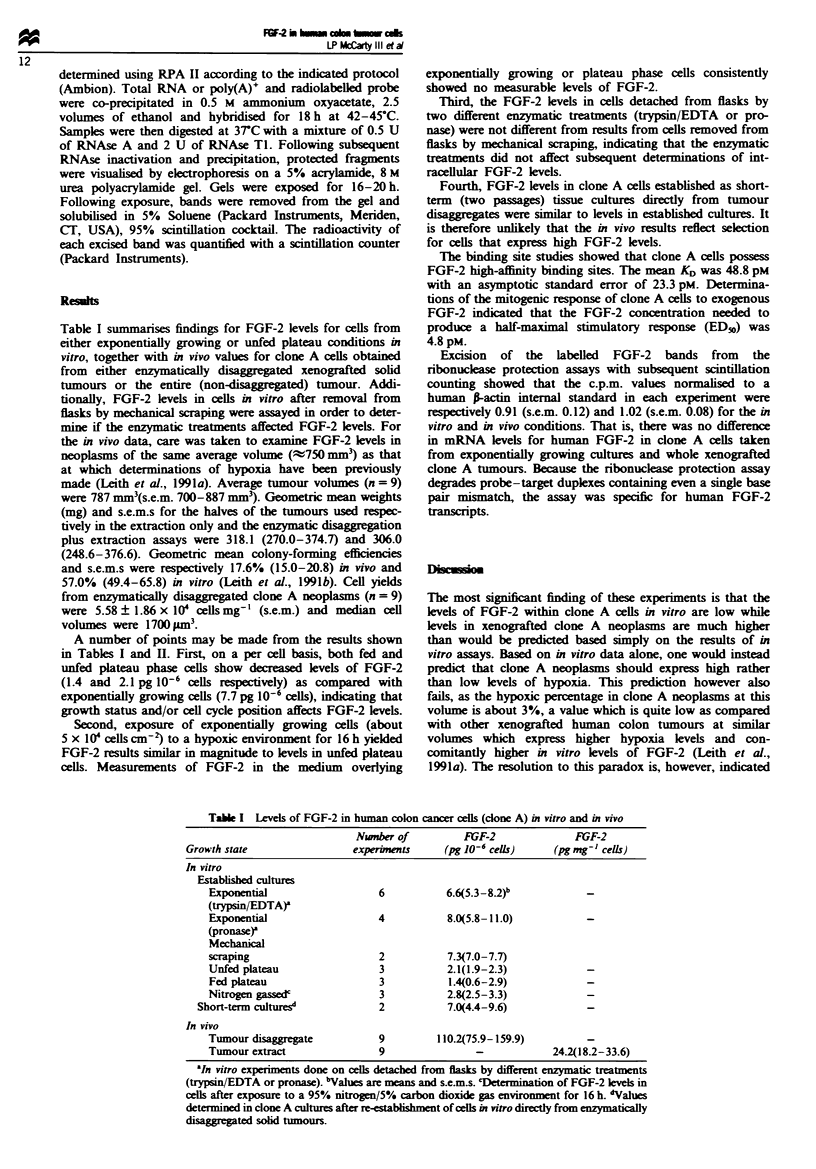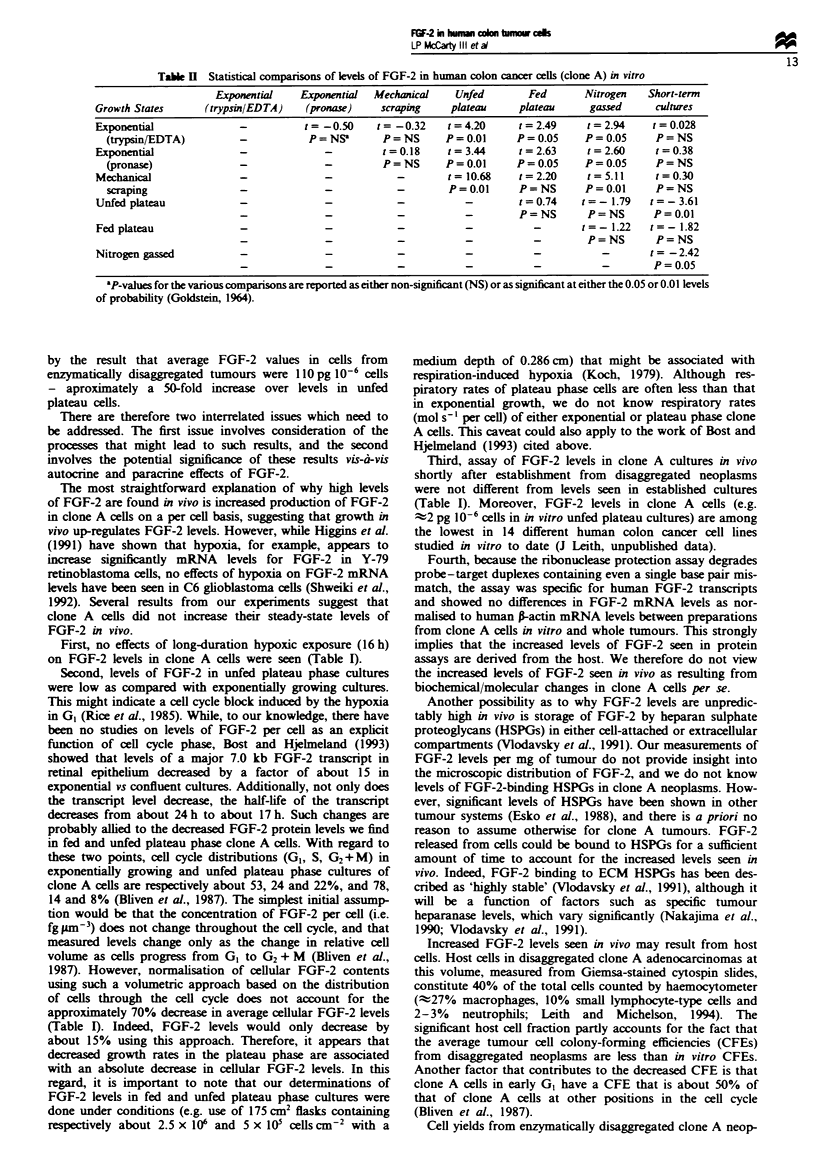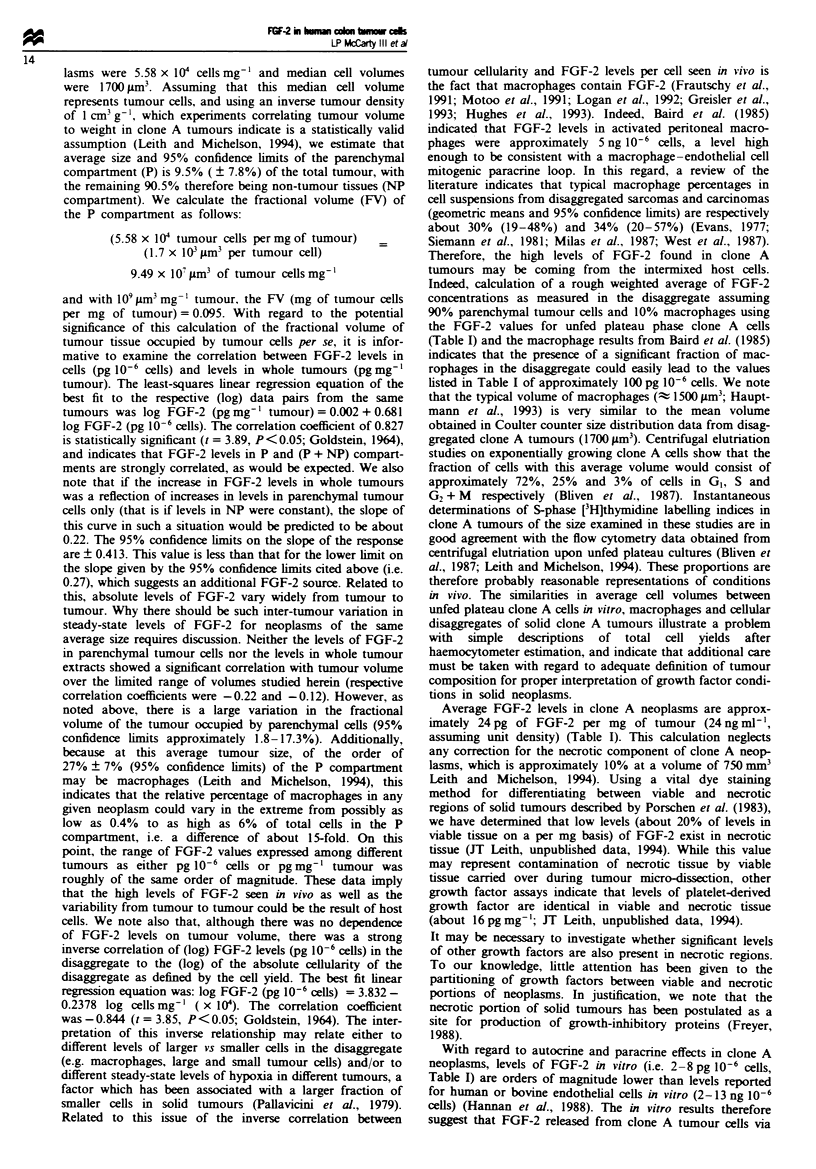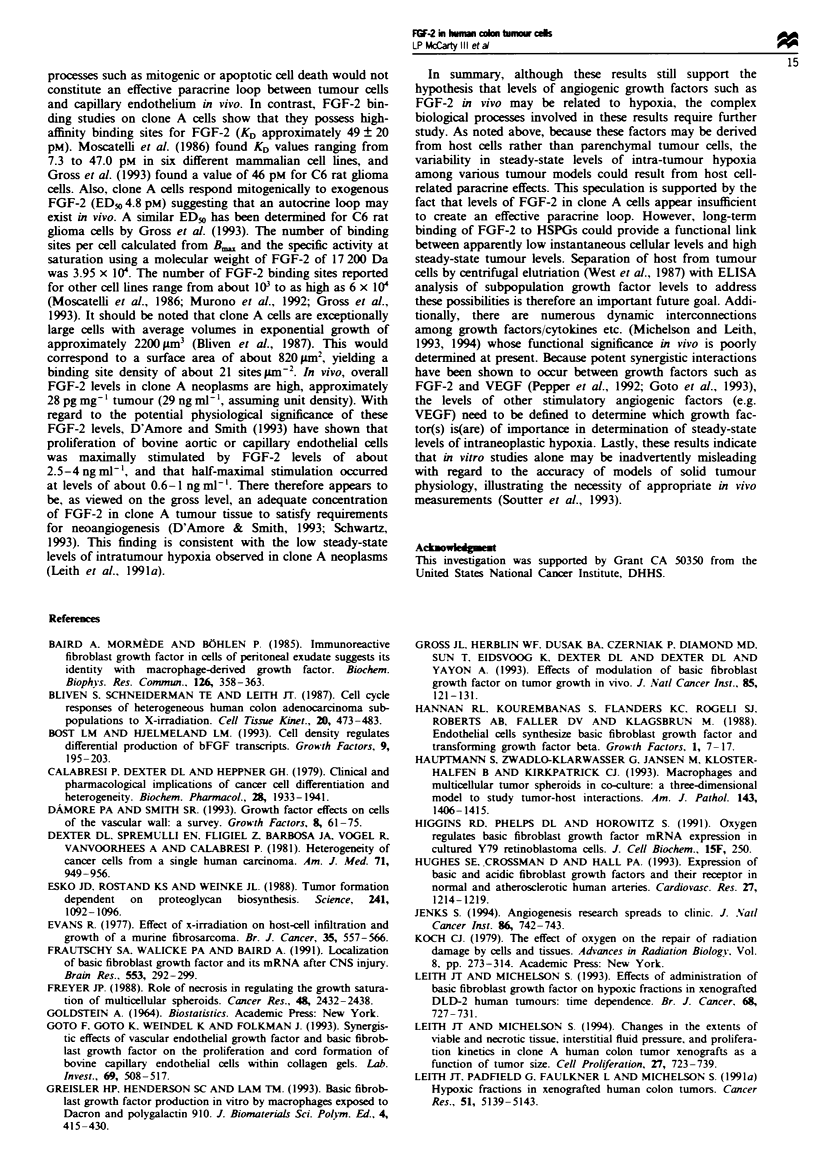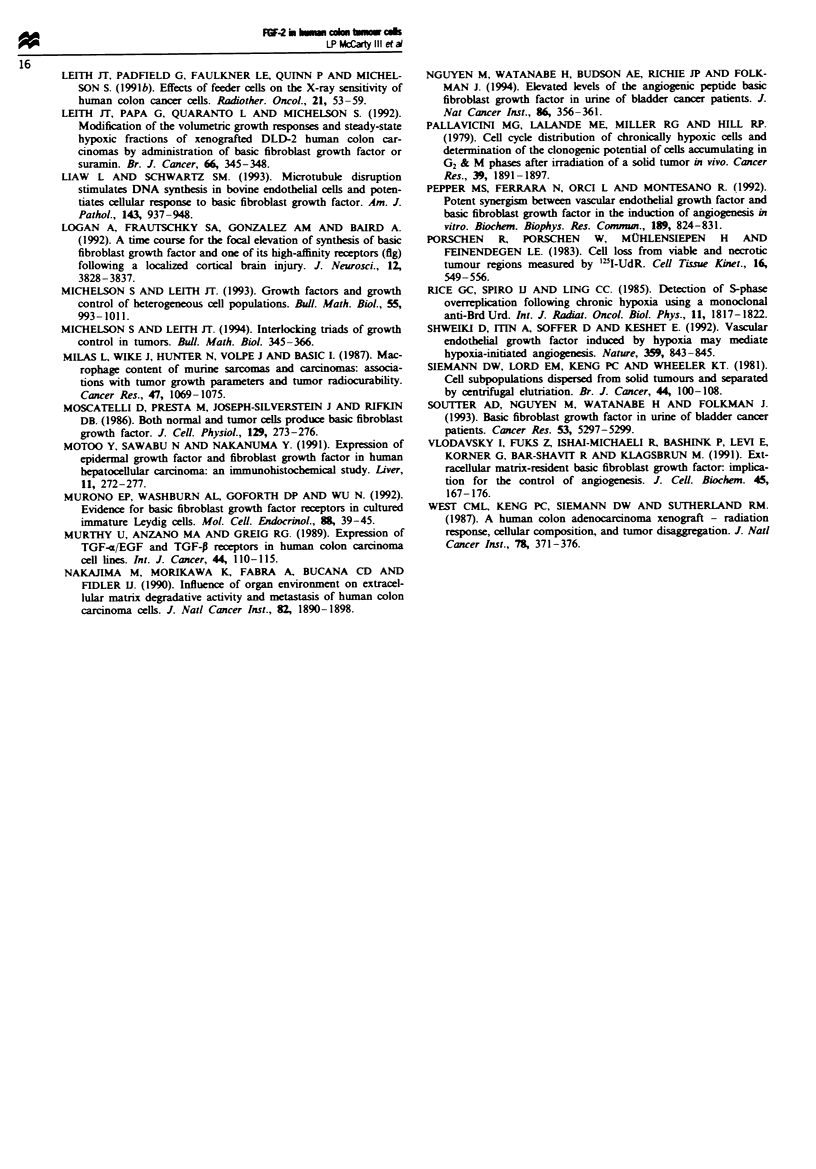# Comparison of basic fibroblast growth factor levels in clone A human colon cancer cells in vitro with levels in xenografted tumours.

**DOI:** 10.1038/bjc.1995.269

**Published:** 1995-07

**Authors:** L. P. McCarty, S. M. Karr, B. Z. Harris, S. G. Michelson, J. T. Leith

**Affiliations:** Department of Radiation Medicine, Brown University School of Medicine, Providence, Rhode Island 02912, USA.

## Abstract

We measured levels of basic fibroblast growth factor (FGF-2) in human colon cancer cells (clone A) in vitro and in xenografted solid tumours using a commercial enzyme-linked immunoassay. In Vitro, levels in unfed plateau phase or exponentially growing cells were low, averaging respectively about 2 and 8 pg 10(-6) cells. However, when solid tumours (average volumes 787 mm3) were cut into halves and either enzymatically disaggregated to obtain a cellular fraction or extracted in toto, levels were much higher. In the cellular fraction, values averaged 110 pg 10(-6) cells, while in whole tumour extracts, average values were 24 pg mg-1 tumour tissue. These results indicate that growth factor levels in solid neoplasms may differ markedly from those predicted from in vitro measurements. We hypothesise that the apparent increase in FGF-2 levels in vivo results primarily from the presence of a significant fraction of host cells (in particular, macrophages, which may contain high levels of FGF-2) within xenografted clone A neoplasms.


© 1995 Stockton Press All rights reserved 0007-0920/95 $12.00

### Comparison of basic fibroblast growth factor levels in clone A human colon cancer cells *in vitro* with levels in xenografted tumours

LP McCarty III[1], SM Karr[1], BZ Harris[1], SG Michelson[2] and JT Leith[1]

[1]*Radiation Research Laboratories, Department of Radiation Medicine, Brown University School of Medicine, Providence, Rhode Island 02912, USA;* [2]*Syntex Corporation, Department of Biomathematics, Palo Alto, California 94303, USA.*

**Summary** We measured levels of basic fibroblast growth factor (FGF-2) in human colon cancer cells (clone A) *in vitro* and in xenografted solid tumours using a commercial enzyme-linked immunoassay. *In Vitro*, levels in unfed plateau phase or exponentially growing cells were low, averaging respectively about 2 and 8 pg $10^{-6}$ cells. However, when solid tumours (average volumes 787 $mm^3$) were cut into halves and either enzymatically disaggregated to obtain a cellular fraction or extracted *in toto*, levels were much higher. In the cellular fraction, values averaged 110 pg $10^{-6}$ cells, while in whole tumour extracts, average values were 24 pg $mg^{-1}$ tumour tissue. These results indicate that growth factor levels in solid neoplasms may differ markedly from those predicted from *in vitro* measurements. We hypothesise that the apparent increase in FGF-2 levels *in vivo* results primarily from the presence of a significant fraction of host cells (in particular, macrophages, which may contain high levels of FGF-2) within xenografted clone A neoplasms.

**Keywords:** basic fibroblast growth factor; xenografted tumours; hypoxia; host cells

Angiogenic peptides such as basic fibroblast growth factor (FGF-2) appear to have substantial roles in the regulation of tumour growth (Soutter *et al.*, 1993; Nguyen *et al.*, 1994) and therefore may be considered targets for anti-angiogenesis cancer treatments, possibly in conjunction with chemo- or radiotherapy (Jenks, 1994). Such combined therapies, however, may present subtle problems. For example, we have shown that treatment of xenografted human tumours with exogenous FGF-2 increases growth rates and decreases hypoxia levels (Leith *et al.*, 1992; Leith and Mitchelson, 1993). Conversely, administration of suramin, a growth factor receptor-blocking agent, slows tumour growth while increasing steady-state levels of hypoxia (Leith *et al.*, 1992). Because hypoxic cells are radiation resistant, strategies that rely upon blocking neovascularisation could produce an unfavourable radiotherapeutic situation. With this caveat in mind, we investigated some aspects of tumour biology related to the feasibility of a radiotherapeutic/anti-angiogenesis approach.

We have previously determined steady-state levels of intratumour hypoxia in a large number of xenografted solid human colon cancers in nude mice, and demonstrated that at similar volumes percentages of hypoxia varied from less than 1% to over 80% (Leith *et al.*, 1991*a*). These widely divergent results led to the hypothesis that variabilaity in hypoxia expression might be inversely related to intraneoplastic levels of angiogenic factors such as FGF-2. According to this logic, cells that produce high levels of FGF-2 would generate well-vascularised solid tumours. Then, as a consequence of this putative well-vascularized situation, such neoplasms should in turn express low steady-state levels of hypoxia. Therefore, we determined levels of FGF-2 both *in vitro* and *in vivo* for the human colon tumour cell line, clone A, chosen because hypoxia levels in these neoplasms are low (≈3%) (Leith *et al.*, 1991*a*). Based on our hypothesis, we a priori predicted that high cellular levels of FGF-2 would be present. Unexpectedly, however, we found that FGF-2 levels *in vitro* on a per cell basis were not only very low, but were also several orders of magnitude less than *in vivo* levels.

Correspondence: JT Leith, Radiation Research Laboratories, Box G, Room B-004, Brown University School of Medicine, Providence RI 02912, USA

Received 16 September 1994; revised 17 January 1995; accepted 21 February 1995

### Materials and methods

### Cell line

The clone A cell line was established in 1978, at the Roger Williams Cancer Center, Providence, RI, USA, from a biopsy specimen from a male patient with a poorly to moderately differentiated primary colonic adenocarcinoma. The primary tumour was found to be heterogeneous, and two cell lines (clones A and D) which differ significantly in morphology and chromosomal number were established. Details on subpopulation isolation have been published (Calabresi *et al.*, 1979; Dexter *et al.*, 1981). For these experiments, stock cells stored in liquid nitrogen were grown in RPMI-1640 medium containing 10% fetal bovine serum (FBS), 1% sodium bicarbonate, 1% anti-PPLO reagent, 1% 4-(2-hydroxyethyl)-1-piperazine-ethanesulphonic acid buffer and 0.5% gentamicin (all reagents from Grand Island Biological, Grand Island, NY, USA).

### Mice and production of xenograft tumours

Young adult male nude mice were obtained from the Charles River Breeding Laboratories, North Wilmington, MA, USA. Mice were housed, ten per large cage, with dust covers, in a dedicated room in a laminar flow hood (Thoren Industries, King of Prussia, PA, USA). Mice were quarantined for 1 week and were ear tagged for identification. To produce tumours (one per animal), clone A cells were trypsinised (0.05% trypsin, 0.54 mM EDTA) from exponentially growing cultures, and resuspended as single cells in Hanks' basic salt solution (HBSS) at a concentration of $5 \times 10^7$ cells $ml^{-1}$. A 0.2 ml volume of the cell suspension was injected into the right flank region of each mouse.

### Determination of in vitro and in vivo levels of FGF-2

*In vitro*, clone A cells were enzymatically removed from either exponentially growing, unfed or fed plateau cultures (cell densities respectively $3 \times 10^4$, $5 \times 10^5$ and $2.5 \times 10^6$ cells $cm^{-2}$), using either trypsin (0.05% trypsin, 0.54 mM EDTA) or pronase (0.25% nuclease-free neutral protease (Calbiochem, San Diego, CA, USA). Cultures were initiated at a density of $10^4$ cells $cm^{-2}$, and were respectively assayed for FGF-2 levels at 3, 8 and 11 days post-seeding. For the fed plateau cultures, the medium (50 ml 175 $cm^{-2}$ flask) was changed daily. The two different enzymes were compared to see if the specific protease used affected FGF-2 levels. Additionally, exponentially growing cells were removed from flasks by mechanical scraping as a control to determine if enzymatic removal in general affected FGF-2 levels. Cells were centrifuged, resuspended and washed twice in ice-cold Dulbecco's phosphate-buffered saline (DPBS). Cell lysates were prepared by resuspension of cells (final concentration 2–5 × $10^7$ cells $ml^{-1}$) in an extraction buffer (4°C) containing 10 mM Tris–HCl (pH7.0), 2M sodium chloride, 0.02% (3-[(3-cholamidopropyl)-dimethylammonio]- 1-propanesulphonate) (CHAPS, Sigma, St. Louis, MO, USA) and protease inhibitors [2 μg $ml^{-1}$ bestatin, pepstatin, and elastatinal, 400 μM leupeptin (Sigma) and 2 μg $ml^{-1}$ phenylmethylsulphonyl fluoride (Boehringer Mannheim Biochemicals, Indianapolis, IN, USA)]. The lysate was sonicated (4–15 s bursts on ice, 70–80% power, Heat Systems sonicator, model 225-R, Farmingdale, NY, USA), and centrifuged (4°C, 26 900 *g*, 2 h, Beckman J2-21 centrifuge, Beckman Instruments, Palo Alto, CA, USA to produce the cell extract supernatant for assay.

*In vivo*, each tumour was sterilely removed and cut into approximately equal halves. Both halves were then weighed and minced using opposed scalpel blades into fragments of less than 1 $mm^3$. One of the halves was disaggregated into single cells in a stirred (40 min, 37°C) enzyme cocktail consisting of 0.2% RNAse-free DNAse (Sigma), 0.25% collagenase (Boehringer Mannheim), 0.25% nuclease-free neutral protease (Calbiochem) in RPMI-1640. This disaggregate was then sieved through an 80 $\mu m^2$ rectangular steel mesh, centrifuged (1000 r.p.m., 10 min, 4°C), resuspended and counted by haemocytometer. The other tumour half was not enzymatically disaggregated. Instead, the mince fragments were extracted *in toto* in complete lysis buffer. Therefore, assays of the two halves of each neoplasm represent directly comparable levels of FGF-2 in the tumour parenchyma (P compartment, enzyme disaggregate) and in the parenchymal plus non-parenchymal compartments (P + NP, *in toto* extraction).

FGF-levels were determined in replicate from each *in vitro* or *in vivo* sample supernatant using an enzyme-linked immunoassay (ELISA) kit (R&D Systems, Minneapolis, MN, USA). Means and s.e.m.s were determined from logs of individual determinations. Protein levels in samples were determined using the Bradford assay (BioRad, Richmond CA, USA).

### *Determination of* $K_D$ *and* $B_{max}$ *for FGF-basic high-affinity binding sites*

Our assay was adapted from previously described protocols (Moscatelli, 1986; Murthy *et al*., 1989). Cells from stock cultures were washed once with DPBS, trypsinised, counted by haemocytometer and seeded in 24 well culture plates (Costar, Cambridge, MA, USA) at a density of 6 × $10^4$ cells per well. Forty-eight hours after seeding and 2 h before the experiment, cells were washed twice with 0.5 ml of ice-cold DPBS and incubated at 37°C in 0.5 ml of Ex-Cell (JR Biosciences, Lenexa, KS, USA) with 0.1% bovine serum albumin (BSA) (Calbiochem) and 25 mM Hepes, pH 7.5. Cells were washed twice with 0.5 ml of ice-cold DPBS, and an appropriate concentration of $^{125}I$-labelled recombinant human FGF-2 (specific activity 124 μCi $\mu g^{-1}$) (New England Nuclear, Boston, MA, USA) in Ex-Cell (0.1% BSA, 25 mM Hepes, pH 7.5) was added to each well (each concentration was assayed in duplicate) in a final volume of 400 μl. After addition of labelled FGF-2, cells were incubated for 2 h at 4°C on an orbital shaker to permit saturation of binding sites. Following incubation, cells were washed twice with 0.5 ml of ice-cold DPBS and cell-bound $^{125}I$-labelled FGF-2 was analysed by solubilising cells with 1 ml per well 0.5% Triton X-100 in 0.1 M sodium phosphate, pH 8.1 (20 min, room temperature) on an orbital shaker. Cell-bound $^{125}I$-labelled FGF-2 (c.p.m.) was determined using a Beckman Gamma 4000 gamma-counter (Beckman Instruments), which has a counting efficiency for $^{125}I$ of 0.77 c.p.m./d.p.m. Non-specific binding was determined using a 250–300 molar excess of recombinant human FGF-2 (R&D Systems) in addition to appropriate concentrations of $^{125}I$-labelled FGF-2. $K_D$ and $B_{max}$ were determined using a non-linear regression fit of total binding data from three independent experiments to the model $X(B_{max})$ $K_D + X$ (NSB) where $X$ is the concentration of radiolabelled ligand and NSB is the non-specific binding. In each experiment representative wells were trypsinised and counted by haemocytometer to allow calculation of the number of binding sites per cell.

### *Cell proliferation studies*

Mitogenic responses to exogenously administered FGF-2 were measured using an MTT colorimetric assay. For the MTT assay, cells were seeded into 24 well plates (Costar) at a density of 1.3 × $10^5$ cells per well in 2 ml of complete RPMI-1640 medium containing 10% FBS and incubated at 37°C for 48 h. Cells were then washed once with DPBS preheated to 37°C and incubated in 1 ml of Ex-Cell 300, chemically defined medium at 37°C for 24 h. Following the incubation in defined medium, varying concentrations of FGF-2 or vascular endothelial growth factor (VEGF) were added to each well in a volume of 100 μl of sterile deionised water with 0.02% CHAPS (Sigma) detergent. After the addition of FGF-2, cells (with appropriate negative controls) were incubated at 37°C for an additional 48 h. MTT reagent was then added to each well at a final concentration of 0.5 mg $ml^{-1}$, and cells were incubated at 37°C for 4 h. Next, cells were solubilised overnight using 1 ml of 10% SDS in 0.01 M hydrochloric acid. Aliquots from each well were transferred to a 96 well ELISA plate (Costar) and read at 590 nm with a microplate reader (Bio Tek Instruments, Woonski, VT, USA). Samples were assayed in triplicate.

### *Ribonuclease protection assay*

Poly(A)$^+$ RNA was prepared using the Fast Track mRNA isolation system (Invitrogen, San Diego, CA, USA). Polymerase chain reaction (PCR)-specific primers were designed from sequences obtained from GenBank and were subjected to presynthesis analysis for dimerisation and specificity with the aid of Amplify v1.2 (Dr Bill Engels, University of Wisconsin). Primers were synthesised by Oligos, Etc. (Wilsonville, OR, USA). DNA templates incorporating promoters for phage polymerases are necessary for production of an antisense-labelled probe for use in ribonuclease protection assay. Synthesis of the required template by PCR was accomplished using a reverse primer that incorporated a T7 phage promoter sequence upstream of the primer binding region. FGF-2 template DNA was made from a plasmid, pHFL1-7, containing an 800 bp fragment of the cDNA for FGF-2 (courtesy Dr Judith Abraham, Scios Nova, Mountain View, CA, USA). PCR was performed directly on the plasmid. Sequences of FGF-2 primers used in template synthesis were as follows:

5′-CAAGCAGAAGAGAGAGGA-3′(forward)
5′-AATCTCTAATACGACTCACTATAGGGAGG
CTCTTAGCACACATTGG-3′ (reverse)

*In vitro* transcription was performed using MAXIscript (Ambion, Austin, TX, USA). Template DNA was incubated at ambient temperature for 60 min with 0.5 mM ATP, CTP and GTP, 10 mM DTT, 0.5 mCi of[α-$^{32}P$]UTP at 800 Ci $mmol^{-1}$, 12.5 U of human placental ribonuclease inhibitor and 10 U of T7 RNA polymerase. A lower specific activity human β-actin control template was synthesised with 0.1 mCi of [α-$^{32}P$]UTP and 0.1 mM UTP. Transcription was terminated by the addition of 2 U of DNAse I followed by a 15 min incubation at 37°C. Radiolabelled probe was purified by electrophoresis on a 5% acrylamide, 8 M urea polyacrylamide gel. The probe hu-FGF-2 was synthesised at approximately 1.3 × $10^9$ c.p.m. $mg^{-1}$, and the probe for human β-actin was synthesised at approximately 1.6 × $10^3$ c.p.m. $mg^{-1}$.

Numbers of transcripts for FGF-2 *in vitro* and *in vivo* were determined using RPA II according to the indicated protocol (Ambion). Total RNA or poly(A)$^+$ and radiolabelled probe were co-precipitated in 0.5 M ammonium oxyacetate, 2.5 volumes of ethanol and hybridised for 18 h at 42–45°C. Samples were then digested at 37°C with a mixture of 0.5 U of RNAse A and 2 U of RNAse T1. Following subsequent RNAse inactivation and precipitation, protected fragments were visualised by electrophoresis on a 5% acrylamide, 8 M urea polyacrylamide gel. Gels were exposed for 16–20 h. Following exposure, bands were removed from the gel and solubilised in 5% Soluene (Packard Instruments, Meriden, CT, USA), 95% scintillation cocktail. The radioactivity of each excised band was quantified with a scintillation counter (Packard Instruments).

### Results

Table I summarises findings for FGF-2 levels for cells from either exponentially growing or unfed plateau conditions *in vitro*, together with *in vivo* values for clone A cells obtained from either enzymatically disaggregated xenografted solid tumours or the entire (non-disaggregated) tumour. Additionally, FGF-2 levels in cells *in vitro* after removal from flasks by mechanical scraping were assayed in order to determine if the enzymatic treatments affected FGF-2 levels. For the *in vivo* data, care was taken to examine FGF-2 levels in neoplasms of the same average volume ($\approx$750 mm$^3$) as that at which determinations of hypoxia have been previously made (Leith *et al.*, 1991*a*). Average tumour volumes ($n = 9$) were 787 mm$^3$(s.e.m. 700–887 mm$^3$). Geometric mean weights (mg) and s.e.m.s for the halves of the tumours used respectively in the extraction only and the enzymatic disaggregation plus extraction assays were 318.1 (270.0–374.7) and 306.0 (248.6–376.6). Geometric mean colony-forming efficiencies and s.e.m.s were respectively 17.6% (15.0–20.8) *in vivo* and 57.0% (49.4–65.8) *in vitro* (Leith *et al.*, 1991*b*). Cell yields from enzymatically disaggregated clone A neoplasms ($n = 9$) were $5.58 \pm 1.86 \times 10^4$ cells mg$^{-1}$ (s.e.m.) and median cell volumes were 1700 $\mu$m$^3$.

A number of points may be made from the results shown in Tables I and II. First, on a per cell basis, both fed and unfed plateau phase cells show decreased levels of FGF-2 (1.4 and 2.1 pg $10^{-6}$ cells respectively) as compared with exponentially growing cells (7.7 pg $10^{-6}$ cells), indicating that growth status and/or cell cycle position affects FGF-2 levels.

Second, exposure of exponentially growing cells (about $5 \times 10^4$ cells cm$^{-2}$) to a hypoxic environment for 16 h yielded FGF-2 results similar in magnitude to levels in unfed plateau cells. Measurements of FGF-2 in the medium overlying exponentially growing or plateau phase cells consistently showed no measurable levels of FGF-2.

Third, the FGF-2 levels in cells detached from flasks by two different enzymatic treatments (trypsin/EDTA or pronase) were not different from results from cells removed from flasks by mechanical scraping, indicating that the enzymatic treatments did not affect subsequent determinations of intracellular FGF-2 levels.

Fourth, FGF-2 levels in clone A cells established as short-term (two passages) tissue cultures directly from tumour disaggregates were similar to levels in established cultures. It is therefore unlikely that the *in vivo* results reflect selection for cells that express high FGF-2 levels.

The binding site studies showed that clone A cells possess FGF-2 high-affinity binding sites. The mean $K_D$ was 48.8 pM with an asymptotic standard error of 23.3 pM. Determinations of the mitogenic response of clone A cells to exogenous FGF-2 indicated that the FGF-2 concentration needed to produce a half-maximal stimulatory response ($ED_{50}$) was 4.8 pM.

Excision of the labelled FGF-2 bands from the ribonuclease protection assays with subsequent scintillation counting showed that the c.p.m. values normalised to a human $\beta$-actin internal standard in each experiment were respectively 0.91 (s.e.m. 0.12) and 1.02 (s.e.m. 0.08) for the *in vitro* and *in vivo* conditions. That is, there was no difference in mRNA levels for human FGF-2 in clone A cells taken from exponentially growing cultures and whole xenografted clone A tumours. Because the ribonuclease protection assay degrades probe–target duplexes containing even a single base pair mismatch, the assay was specific for human FGF-2 transcripts.

### Discussion

The most significant finding of these experiments is that the levels of FGF-2 within clone A cells *in vitro* are low while levels in xenografted clone A neoplasms are much higher than would be predicted based simply on the results of *in vitro* assays. Based on *in vitro* data alone, one would instead predict that clone A neoplasms should express high rather than low levels of hypoxia. This prediction however also fails, as the hypoxic percentage in clone A neoplasms at this volume is about 3%, a value which is quite low as compared with other xenografted human colon tumours at similar volumes which express higher hypoxia levels and concomitantly higher *in vitro* levels of FGF-2 (Leith *et al.*, 1991*a*). The resolution to this paradox is, however, indicated

**Table I** Levels of FGF-2 in human colon cancer cells (clone A) *in vitro* and *in vivo*

| *Growth state* | *Number of experiments* | *FGF-2 (pg $10^{-6}$ cells)* | *FGF-2 (pg mg$^{-1}$ cells)* |
|---|---|---|---|
| *In vitro* | | | |
| Established cultures | | | |
| Exponential (trypsin/EDTA)[a] | 6 | 6.6(5.3–8.2)[b] | – |
| Exponential (pronase)[a] | 4 | 8.0(5.8–11.0) | – |
| Mechanical scraping | 2 | 7.3(7.0–7.7) | |
| Unfed plateau | 3 | 2.1(1.9–2.3) | – |
| Fed plateau | 3 | 1.4(0.6–2.9) | – |
| Nitrogen gassed[c] | 3 | 2.8(2.5–3.3) | – |
| Short-term cultures[d] | 2 | 7.0(4.4–9.6) | – |
| *In vivo* | | | |
| Tumour disaggregate | 9 | 110.2(75.9–159.9) | – |
| Tumour extract | 9 | – | 24.2(18.2–33.6) |

[a]*In vitro* experiments done on cells detached from flasks by different enzymatic treatments (trypsin/EDTA or pronase). [b]Values are means and s.e.m.s. [c]Determination of FGF-2 levels in cells after exposure to a 95% nitrogen/5% carbon dioxide gas environment for 16 h. [d]Values determined in clone A cultures after re-establishment of cells *in vitro* directly from enzymatically disaggregated solid tumours.

**Table II** Statistical comparisons of levels of FGF-2 in human colon cancer cells (clone A) *in vitro*

| *Growth States* | *Exponential (trypsin/EDTA)* | *Exponential (pronase)* | *Mechanical scraping* | *Unfed plateau* | *Fed plateau* | *Nitrogen gassed* | *Short-term cultures* |
|---|---|---|---|---|---|---|---|
| Exponential | – | $t=-0.50$ | $t=-0.32$ | $t=4.20$ | $t=2.49$ | $t=2.94$ | $t=0.028$ |
| (trypsin/EDTA) | – | $P=$ NS[a] | $P=$ NS | $P=0.01$ | $P=0.05$ | $P=0.05$ | $P=$ NS |
| Exponential | – | – | $t=0.18$ | $t=3.44$ | $t=2.63$ | $t=2.60$ | $t=0.38$ |
| (pronase) | – | – | $P=$ NS | $P=0.01$ | $P=0.05$ | $P=0.05$ | $P=$ NS |
| Mechanical | – | – | – | $t=10.68$ | $t=2.20$ | $t=5.11$ | $t=0.30$ |
| scraping | – | – | – | $P=0.01$ | $P=$ NS | $P=0.01$ | $P=$ NS |
| Unfed plateau | – | – | – | – | $t=0.74$ | $t=-1.79$ | $t=-3.61$ |
| | – | – | – | – | $P=$ NS | $P=$ NS | $P=0.01$ |
| Fed plateau | – | – | – | – | – | $t=-1.22$ | $t=-1.82$ |
| | – | – | – | – | – | $P=$ NS | $P=$ NS |
| Nitrogen gassed | – | – | – | – | – | – | $t=-2.42$ |
| | – | – | – | – | – | – | $P=0.05$ |

[a]*P*-values for the various comparisons are reported as either non-significant (NS) or as significant at either the 0.05 or 0.01 levels of probability (Goldstein, 1964).

by the result that average FGF-2 values in cells from enzymatically disaggregated tumours were 110 pg $10^{-6}$ cells – aproximately a 50-fold increase over levels in unfed plateau cells.

There are therefore two interrelated issues which need to be addressed. The first issue involves consideration of the processes that might lead to such results, and the second involves the potential significance of these results *vis-à-vis* autocrine and paracrine effects of FGF-2.

The most straightforward explanation of why high levels of FGF-2 are found *in vivo* is increased production of FGF-2 in clone A cells on a per cell basis, suggesting that growth *in vivo* up-regulates FGF-2 levels. However, while Higgins *et al.* (1991) have shown that hypoxia, for example, appears to increase significantly mRNA levels for FGF-2 in Y-79 retinoblastoma cells, no effects of hypoxia on FGF-2 mRNA levels have been seen in C6 glioblastoma cells (Shweiki *et al.*, 1992). Several results from our experiments suggest that clone A cells did not increase their steady-state levels of FGF-2 *in vivo*.

First, no effects of long-duration hypoxic exposure (16 h) on FGF-2 levels in clone A cells were seen (Table I).

Second, levels of FGF-2 in unfed plateau phase cultures were low as compared with exponentially growing cultures. This might indicate a cell cycle block induced by the hypoxia in $G_1$ (Rice *et al.*, 1985). While, to our knowledge, there have been no studies on levels of FGF-2 per cell as an explicit function of cell cycle phase, Bost and Hjelmeland (1993) showed that levels of a major 7.0 kb FGF-2 transcript in retinal epithelium decreased by a factor of about 15 in exponential *vs* confluent cultures. Additionally, not only does the transcript level decrease, the half-life of the transcript decreases from about 24 h to about 17 h. Such changes are probably allied to the decreased FGF-2 protein levels we find in fed and unfed plateau phase clone A cells. With regard to these two points, cell cycle distributions ($G_1$, S, $G_2+M$) in exponentially growing and unfed plateau phase cultures of clone A cells are respectively about 53, 24 and 22%, and 78, 14 and 8% (Bliven *et al.*, 1987). The simplest initial assumption would be that the concentration of FGF-2 per cell (i.e. fg $\mu m^{-3}$) does not change throughout the cell cycle, and that measured levels change only as the change in relative cell volume as cells progress from $G_1$ to $G_2+M$ (Bliven *et al.*, 1987). However, normalisation of cellular FGF-2 contents using such a volumetric approach based on the distribution of cells through the cell cycle does not account for the approximately 70% decrease in average cellular FGF-2 levels (Table I). Indeed, FGF-2 levels would only decrease by about 15% using this approach. Therefore, it appears that decreased growth rates in the plateau phase are associated with an absolute decrease in cellular FGF-2 levels. In this regard, it is important to note that our determinations of FGF-2 levels in fed and unfed plateau phase cultures were done under conditions (e.g. use of 175 $cm^2$ flasks containing respectively about $2.5\times10^6$ and $5\times10^5$ cells $cm^{-2}$ with a medium depth of 0.286 cm) that might be associated with respiration-induced hypoxia (Koch, 1979). Although respiratory rates of plateau phase cells are often less than that in exponential growth, we do not know respiratory rates (mol $s^{-1}$ per cell) of either exponential or plateau phase clone A cells. This caveat could also apply to the work of Bost and Hjelmeland (1993) cited above.

Third, assay of FGF-2 levels in clone A cultures *in vivo* shortly after establishment from disaggregated neoplasms were not different from levels seen in established cultures (Table I). Moreover, FGF-2 levels in clone A cells (e.g. $\approx$2 pg $10^{-6}$ cells in *in vitro* unfed plateau cultures) are among the lowest in 14 different human colon cancer cell lines studied *in vitro* to date (J Leith, unpublished data).

Fourth, because the ribonuclease protection assay degrades probe–target duplexes containing even a single base pair mismatch, the assay was specific for human FGF-2 transcripts and showed no differences in FGF-2 mRNA levels as normalised to human $\beta$-actin mRNA levels between preparations from clone A cells *in vitro* and whole tumours. This strongly implies that the increased levels of FGF-2 seen in protein assays are derived from the host. We therefore do not view the increased levels of FGF-2 seen *in vivo* as resulting from biochemical/molecular changes in clone A cells *per se*.

Another possibility as to why FGF-2 levels are unpredictably high *in vivo* is storage of FGF-2 by heparan sulphate proteoglycans (HSPGs) in either cell-attached or extracellular compartments (Vlodavsky *et al.*, 1991). Our measurements of FGF-2 levels per mg of tumour do not provide insight into the microscopic distribution of FGF-2, and we do not know levels of FGF-2-binding HSPGs in clone A neoplasms. However, significant levels of HSPGs have been shown in other tumour systems (Esko *et al.*, 1988), and there is *a priori* no reason to assume otherwise for clone A tumours. FGF-2 released from cells could be bound to HSPGs for a sufficient amount of time to account for the increased levels seen *in vivo*. Indeed, FGF-2 binding to ECM HSPGs has been described as 'highly stable' (Vlodavsky *et al.*, 1991), although it will be a function of factors such as specific tumour heparanase levels, which vary significantly (Nakajima *et al.*, 1990; Vlodavsky *et al.*, 1991).

Increased FGF-2 levels seen *in vivo* may result from host cells. Host cells in disaggregated clone A adenocarcinomas at this volume, measured from Giemsa-stained cytospin slides, constitute 40% of the total cells counted by haemocytometer ($\approx$27% macrophages, 10% small lymphocyte-type cells and 2–3% neutrophils; Leith and Michelson, 1994). The significant host cell fraction partly accounts for the fact that the average tumour cell colony-forming efficiencies (CFEs) from disaggregated neoplasms are less than *in vitro* CFEs. Another factor that contributes to the decreased CFE is that clone A cells in early $G_1$ have a CFE that is about 50% of that of clone A cells at other positions in the cell cycle (Bliven *et al.*, 1987).

Cell yields from enzymatically disaggregated clone A neoplasms were $5.58 \times 10^4$ cells $mg^{-1}$ and median cell volumes were 1700 $\mu m^3$. Assuming that this median cell volume represents tumour cells, and using an inverse tumour density of 1 $cm^3\ g^{-1}$, which experiments correlating tumour volume to weight in clone A tumours indicate is a statistically valid assumption (Leith and Michelson, 1994), we estimate that average size and 95% confidence limits of the parenchymal compartment (P) is 9.5% ( ± 7.8%) of the total tumour, with the remaining 90.5% therefore being non-tumour tissues (NP compartment). We calculate the fractional volume (FV) of the P compartment as follows:

$$\frac{(5.58 \times 10^4 \text{ tumour cells per mg of tumour})}{(1.7 \times 10^3\ \mu m^3 \text{ per tumour cell})} = 9.49 \times 10^7\ \mu m^3 \text{ of tumour cells mg}^{-1}$$

and with $10^9\ \mu m^3\ mg^{-1}$ tumour, the FV (mg of tumour cells per mg of tumour) = 0.095. With regard to the potential significance of this calculation of the fractional volume of tumour tissue occupied by tumour cells *per se*, it is informative to examine the correlation between FGF-2 levels in cells (pg $10^{-6}$ cells) and levels in whole tumours (pg $mg^{-1}$ tumour). The least-squares linear regression equation of the best fit to the respective (log) data pairs from the same tumours was log FGF-2 (pg $mg^{-1}$ tumour) = 0.002 + 0.681 log FGF-2 (pg $10^{-6}$ cells). The correlation coefficient of 0.827 is statistically significant ($t = 3.89$, $P < 0.05$; Goldstein, 1964), and indicates that FGF-2 levels in P and (P + NP) compartments are strongly correlated, as would be expected. We also note that if the increase in FGF-2 levels in whole tumours was a reflection of increases in levels in parenchymal tumour cells only (that is if levels in NP were constant), the slope of this curve in such a situation would be predicted to be about 0.22. The 95% confidence limits on the slope of the response are ± 0.413. This value is less than that for the lower limit on the slope given by the 95% confidence limits cited above (i.e. 0.27), which suggests an additional FGF-2 source. Related to this, absolute levels of FGF-2 vary widely from tumour to tumour. Why there should be such inter-tumour variation in steady-state levels of FGF-2 for neoplasms of the same average size requires discussion. Neither the levels of FGF-2 in parenchymal tumour cells nor the levels in whole tumour extracts showed a significant correlation with tumour volume over the limited range of volumes studied herein (respective correlation coefficients were −0.22 and −0.12). However, as noted above, there is a large variation in the fractional volume of the tumour occupied by parenchymal cells (95% confidence limits approximately 1.8–17.3%). Additionally, because at this average tumour size, of the order of 27% ± 7% (95% confidence limits) of the P compartment may be macrophages (Leith and Michelson, 1994), this indicates that the relative percentage of macrophages in any given neoplasm could vary in the extreme from possibly as low as 0.4% to as high as 6% of total cells in the P compartment, i.e. a difference of about 15-fold. On this point, the range of FGF-2 values expressed among different tumours as either pg $10^{-6}$ cells or pg $mg^{-1}$ tumour was roughly of the same order of magnitude. These data imply that the high levels of FGF-2 seen *in vivo* as well as the variability from tumour to tumour could be the result of host cells. We note also that, although there was no dependence of FGF-2 levels on tumour volume, there was a strong inverse correlation of (log) FGF-2 levels (pg $10^{-6}$ cells) in the disaggregate to the (log) of the absolute cellularity of the disaggregate as defined by the cell yield. The best fit linear regression equation was: log FGF-2 (pg $10^{-6}$ cells) = 3.832 − 0.2378 log cells $mg^{-1}$ ( × $10^4$). The correlation coefficient was −0.844 ($t = 3.85$, $P < 0.05$; Goldstein, 1964). The interpretation of this inverse relationship may relate either to different levels of larger *vs* smaller cells in the disaggregate (e.g. macrophages, large and small tumour cells) and/or to different steady-state levels of hypoxia in different tumours, a factor which has been associated with a larger fraction of smaller cells in solid tumours (Pallavicini *et al*., 1979). Related to this issue of the inverse correlation between tumour cellularity and FGF-2 levels per cell seen *in vivo* is the fact that macrophages contain FGF-2 (Frautschy *et al*., 1991; Motoo *et al*., 1991; Logan *et al*., 1992; Greisler *et al*., 1993; Hughes *et al*., 1993). Indeed, Baird *et al*. (1985) indicated that FGF-2 levels in activated peritoneal macrophages were approximately 5 ng $10^{-6}$ cells, a level high enough to be consistent with a macrophage–endothelial cell mitogenic paracrine loop. In this regard, a review of the literature indicates that typical macrophage percentages in cell suspensions from disaggregated sarcomas and carcinomas (geometric means and 95% confidence limits) are respectively about 30% (19–48%) and 34% (20–57%) (Evans, 1977; Siemann *et al*., 1981; Milas *et al*., 1987; West *et al*., 1987). Therefore, the high levels of FGF-2 found in clone A tumours may be coming from the intermixed host cells. Indeed, calculation of a rough weighted average of FGF-2 concentrations as measured in the disaggregate assuming 90% parenchymal tumour cells and 10% macrophages using the FGF-2 values for unfed plateau phase clone A cells (Table I) and the macrophage results from Baird *et al*. (1985) indicates that the presence of a significant fraction of macrophages in the disaggregate could easily lead to the values listed in Table I of approximately 100 pg $10^{-6}$ cells. We note that the typical volume of macrophages (≈ 1500 $\mu m^3$; Hauptmann *et al*., 1993) is very similar to the mean volume obtained in Coulter counter size distribution data from disaggregated clone A tumours (1700 $\mu m^3$). Centrifugal elutriation studies on exponentially growing clone A cells show that the fraction of cells with this average volume would consist of approximately 72%, 25% and 3% of cells in $G_1$, S and $G_2 + M$ respectively (Bliven *et al*., 1987). Instantaneous determinations of S-phase [$^3$H]thymidine labelling indices in clone A tumours of the size examined in these studies are in good agreement with the flow cytometry data obtained from centrifugal elutriation upon unfed plateau cultures (Bliven *et al*., 1987; Leith and Michelson, 1994). These proportions are therefore probably reasonable representations of conditions *in vivo*. The similarities in average cell volumes between unfed plateau clone A cells *in vitro*, macrophages and cellular disaggregates of solid clone A tumours illustrate a problem with simple descriptions of total cell yields after haemocytometer estimation, and indicate that additional care must be taken with regard to adequate definition of tumour composition for proper interpretation of growth factor conditions in solid neoplasms.

Average FGF-2 levels in clone A neoplasms are approximately 24 pg of FGF-2 per mg of tumour (24 ng $ml^{-1}$, assuming unit density) (Table I). This calculation neglects any correction for the necrotic component of clone A neoplasms, which is approximately 10% at a volume of 750 $mm^3$ Leith and Michelson, 1994). Using a vital dye staining method for differentiating between viable and necrotic regions of solid tumours described by Porschen *et al*. (1983), we have determined that low levels (about 20% of levels in viable tissue on a per mg basis) of FGF-2 exist in necrotic tissue (JT Leith, unpublished data, 1994). While this value may represent contamination of necrotic tissue by viable tissue carried over during tumour micro-dissection, other growth factor assays indicate that levels of platelet-derived growth factor are identical in viable and necrotic tissue (about 16 pg $mg^{-1}$; JT Leith, unpublished data, 1994).

It may be necessary to investigate whether significant levels of other growth factors are also present in necrotic regions. To our knowledge, little attention has been given to the partitioning of growth factors between viable and necrotic portions of neoplasms. In justification, we note that the necrotic portion of solid tumours has been postulated as a site for production of growth-inhibitory proteins (Freyer, 1988).

With regard to autocrine and paracrine effects in clone A neoplasms, levels of FGF-2 *in vitro* (i.e. 2–8 pg $10^{-6}$ cells, Table I) are orders of magnitude lower than levels reported for human or bovine endothelial cells *in vitro* (2–13 ng $10^{-6}$ cells) (Hannan *et al*., 1988). The *in vitro* results therefore suggest that FGF-2 released from clone A tumour cells via processes such as mitogenic or apoptotic cell death would not constitute an effective paracrine loop between tumour cells and capillary endothelium *in vivo*. In contrast, FGF-2 binding studies on clone A cells show that they possess high-affinity binding sites for FGF-2 ($K_D$ approximately 49 ± 20 pM). Moscatelli *et al.* (1986) found $K_D$ values ranging from 7.3 to 47.0 pM in six different mammalian cell lines, and Gross *et al.* (1993) found a value of 46 pM for C6 rat glioma cells. Also, clone A cells respond mitogenically to exogenous FGF-2 ($ED_{50}$ 4.8 pM) suggesting that an autocrine loop may exist *in vivo*. A similar $ED_{50}$ has been determined for C6 rat glioma cells by Gross *et al.* (1993). The number of binding sites per cell calculated from $B_{max}$ and the specific activity at saturation using a molecular weight of FGF-2 of 17 200 Da was $3.95 \times 10^4$. The number of FGF-2 binding sites reported for other cell lines range from about $10^3$ to as high as $6 \times 10^4$ (Moscatelli *et al.*, 1986; Murono *et al.*, 1992; Gross *et al.*, 1993). It should be noted that clone A cells are exceptionally large cells with average volumes in exponential growth of approximately 2200 $\mu m^3$ (Bliven *et al.*, 1987). This would correspond to a surface area of about 820 $\mu m^2$, yielding a binding site density of about 21 sites $\mu m^{-2}$. *In vivo*, overall FGF-2 levels in clone A neoplasms are high, approximately 28 pg $mg^{-1}$ tumour (29 ng $ml^{-1}$, assuming unit density). With regard to the potential physiological significance of these FGF-2 levels, D'Amore and Smith (1993) have shown that proliferation of bovine aortic or capillary endothelial cells was maximally stimulated by FGF-2 levels of about 2.5–4 ng $ml^{-1}$, and that half-maximal stimulation occurred at levels of about 0.6–1 ng $ml^{-1}$. There therefore appears to be, as viewed on the gross level, an adequate concentration of FGF-2 in clone A tumour tissue to satisfy requirements for neoangiogenesis (D'Amore & Smith, 1993; Schwartz, 1993). This finding is consistent with the low steady-state levels of intratumour hypoxia observed in clone A neoplasms (Leith *et al.*, 1991*a*).

In summary, although these results still support the hypothesis that levels of angiogenic growth factors such as FGF-2 *in vivo* may be related to hypoxia, the complex biological processes involved in these results require further study. As noted above, because these factors may be derived from host cells rather than parenchymal tumour cells, the variability in steady-state levels of intra-tumour hypoxia among various tumour models could result from host cell-related paracrine effects. This speculation is supported by the fact that levels of FGF-2 in clone A cells appear insufficient to create an effective paracrine loop. However, long-term binding of FGF-2 to HSPGs could provide a functional link between apparently low instantaneous cellular levels and high steady-state tumour levels. Separation of host from tumour cells by centrifugal elutriation (West *et al.*, 1987) with ELISA analysis of subpopulation growth factor levels to address these possibilities is therefore an important future goal. Additionally, there are numerous dynamic interconnections among growth factors/cytokines etc. (Michelson and Leith, 1993, 1994) whose functional significance *in vivo* is poorly determined at present. Because potent synergistic interactions have been shown to occur between growth factors such as FGF-2 and VEGF (Pepper *et al.*, 1992; Goto *et al.*, 1993), the levels of other stimulatory angiogenic factors (e.g. VEGF) need to be defined to determine which growth factor(s) is(are) of importance in determination of steady-state levels of intraneoplastic hypoxia. Lastly, these results indicate that *in vitro* studies alone may be inadvertently misleading with regard to the accuracy of models of solid tumour physiology, illustrating the necessity of appropriate *in vivo* measurements (Soutter *et al.*, 1993).

**Acknowledgement**
This investigation was supported by Grant CA 50350 from the United States National Cancer Institute, DHHS.

**References**

BAIRD A, MORMÈDE AND BÖHLEN P. (1985). Immunoreactive fibroblast growth factor in cells of peritoneal exudate suggests its identity with macrophage-derived growth factor. *Biochem. Biophys. Res. Commun.*, **126**, 358–363.

BLIVEN S, SCHNEIDERMAN TE AND LEITH JT. (1987). Cell cycle responses of heterogeneous human colon adenocarcinoma subpopulations to X-irradiation. *Cell Tissue Kinet.*, **20**, 473–483.

BOST LM AND HJELMELAND LM. (1993). Cell density regulates differential production of bFGF transcripts. *Growth Factors*, **9**, 195–203.

CALABRESI P, DEXTER DL AND HEPPNER GH. (1979). Clinical and pharmacological implications of cancer cell differentiation and heterogeneity. *Biochem. Pharmacol.*, **28**, 1933–1941.

DÁMORE PA AND SMITH SR. (1993). Growth factor effects on cells of the vascular wall: a survey. *Growth Factors*, **8**, 61–75.

DEXTER DL, SPREMULLI EN, FLIGIEL Z, BARBOSA JA, VOGEL R, VANVOORHEES A AND CALABRESI P. (1981). Heterogeneity of cancer cells from a single human carcinoma. *Am. J. Med.* **71**, 949–956.

ESKO JD, ROSTAND KS AND WEINKE JL. (1988). Tumor formation dependent on proteoglycan biosynthesis. *Science*, **241**, 1092–1096.

EVANS R. (1977). Effect of x-irradiation on host-cell infiltration and growth of a murine fibrosarcoma. *Br. J. Cancer*, **35**, 557–566.

FRAUTSCHY SA, WALICKE PA AND BAIRD A. (1991). Localization of basic fibroblast growth factor and its mRNA after CNS injury. *Brain Res.*, **553**, 292–299.

FREYER JP. (1988). Role of necrosis in regulating the growth saturation of multicellular spheroids. *Cancer Res.*, **48**, 2432–2438.

GOLDSTEIN A. (1964). *Biostatistics*. Academic Press: New York.

GOTO F, GOTO K, WEINDEL K AND FOLKMAN J. (1993). Synergistic effects of vascular endothelial growth factor and basic fibroblast growth factor on the proliferation and cord formation of bovine capillary endothelial cells within collagen gels. *Lab. Invest.*, **69**, 508–517.

GREISLER HP, HENDERSON SC AND LAM TM. (1993). Basic fibroblast growth factor production in vitro by macrophages exposed to Dacron and polygalactin 910. *J. Biomaterials Sci. Polym. Ed.*, **4**, 415–430.

GROSS JL, HERBLIN WF, DUSAK BA, CZERNIAK P, DIAMOND MD, SUN T, EIDSVOOG K, DEXTER DL AND DEXTER DL AND YAYON A. (1993). Effects of modulation of basic fibroblast growth factor on tumor growth in vivo. *J. Natl Cancer Inst.*, **85**, 121–131.

HANNAN RL, KOUREMBANAS S, FLANDERS KC, ROGELI SJ, ROBERTS AB, FALLER DV AND KLAGSBRUN M. (1988). Endothelial cells synthesize basic fibroblast growth factor and transforming growth factor beta. *Growth Factors*, **1**, 7–17.

HAUPTMANN S, ZWADLO-KLARWASSER G, JANSEN M, KLOSTERHALFEN B AND KIRKPATRICK CJ. (1993). Macrophages and multicellular tumor spheroids in co-culture: a three-dimensional model to study tumor-host interactions. *Am. J. Pathol.* **143**, 1406–1415.

HIGGINS RD, PHELPS DL AND HOROWITZ S. (1991). Oxygen regulates basic fibroblast growth factor mRNA expression in cultured Y79 retinoblastoma cells. *J. Cell Biochem.*, **15F**, 250.

HUGHES SE, CROSSMAN D AND HALL PA. (1993). Expression of basic and acidic fibroblast growth factors and their receptor in normal and atherosclerotic human arteries. *Cardiovasc. Res.* **27**, 1214–1219.

JENKS S. (1994). Angiogenesis research spreads to clinic. *J. Natl Cancer Inst.* **86**, 742–743.

KOCH CJ. (1979). The effect of oxygen on the repair of radiation damage by cells and tissues. *Advances in Radiation Biology*, Vol. 8, pp. 273–314. Academic Press: New York.

LEITH JT AND MICHELSON S. (1993). Effects of administration of basic fibroblast growth factor on hypoxic fractions in xenografted DLD-2 human tumours: time dependence. *Br. J. Cancer*, **68**, 727–731.

LEITH JT AND MICHELSON S. (1994). Changes in the extents of viable and necrotic tissue, interstitial fluid pressure, and proliferation kinetics in clone A human colon tumor xenografts as a function of tumor size. *Cell Proliferation*, **27**, 723–739.

LEITH JT, PADFIELD G, FAULKNER L AND MICHELSON S. (1991*a*) Hypoxic fractions in xenografted human colon tumors. *Cancer Res.*, **51**, 5139–5143.

LEITH JT, PADFIELD G, FAULKNER LE, QUINN P AND MICHELSON S. (1991*b*). Effects of feeder cells on the X-ray sensitivity of human colon cancer cells. *Radiother. Oncol.*, **21**, 53–59.

LEITH JT, PAPA G, QUARANTO L AND MICHELSON S. (1992). Modification of the volumetric growth responses and steady-state hypoxic fractions of xenografted DLD-2 human colon carcinomas by administration of basic fibroblast growth factor or suramin. *Br. J. Cancer*, **66**, 345–348.

LIAW L AND SCHWARTZ SM. (1993). Microtubule disruption stimulates DNA synthesis in bovine endothelial cells and potentiates cellular response to basic fibroblast growth factor. *Am. J. Pathol.*, **143**, 937–948.

LOGAN A, FRAUTSCHKY SA, GONZALEZ AM AND BAIRD A. (1992). A time course for the focal elevation of synthesis of basic fibroblast growth factor and one of its high-affinity receptors (flg) following a localized cortical brain injury. *J. Neurosci.*, **12**, 3828–3837.

MICHELSON S AND LEITH JT. (1993). Growth factors and growth control of heterogeneous cell populations. *Bull. Math. Biol.*, **55**, 993–1011.

MICHELSON S AND LEITH JT. (1994). Interlocking triads of growth control in tumors. *Bull. Math. Biol.* 345–366.

MILAS L, WIKE J, HUNTER N, VOLPE J AND BASIC I. (1987). Macrophage content of murine sarcomas and carcinomas: associations with tumor growth parameters and tumor radiocurability. *Cancer Res.*, **47**, 1069–1075.

MOSCATELLI D, PRESTA M, JOSEPH-SILVERSTEIN J AND RIFKIN DB. (1986). Both normal and tumor cells produce basic fibroblast growth factor. *J. Cell. Physiol.*, **129**, 273–276.

MOTOO Y, SAWABU N AND NAKANUMA Y. (1991). Expression of epidermal growth factor and fibroblast growth factor in human hepatocellular carcinoma: an immunohistochemical study. *Liver*, **11**, 272–277.

MURONO EP, WASHBURN AL, GOFORTH DP AND WU N. (1992). Evidence for basic fibroblast growth factor receptors in cultured immature Leydig cells. *Mol. Cell. Endocrinol.*, **88**, 39–45.

MURTHY U, ANZANO MA AND GREIG RG. (1989). Expression of TGF-$\alpha$/EGF and TGF-$\beta$ receptors in human colon carcinoma cell lines. *Int. J. Cancer*, **44**, 110–115.

NAKAJIMA M, MORIKAWA K, FABRA A, BUCANA CD AND FIDLER IJ. (1990). Influence of organ environment on extracellular matrix degradative activity and metastasis of human colon carcinoma cells. *J. Natl Cancer Inst.*, **82**, 1890–1898.

NGUYEN M, WATANABE H, BUDSON AE, RICHIE JP AND FOLKMAN J. (1994). Elevated levels of the angiogenic peptide basic fibroblast growth factor in urine of bladder cancer patients. *J. Nat Cancer Inst.*, **86**, 356–361.

PALLAVICINI MG, LALANDE ME, MILLER RG AND HILL RP. (1979). Cell cycle distribution of chronically hypoxic cells and determination of the clonogenic potential of cells accumulating in $G_2$ & M phases after irradiation of a solid tumor *in vivo*. *Cancer Res.*, **39**, 1891–1897.

PEPPER MS, FERRARA N, ORCI L AND MONTESANO R. (1992). Potent synergism between vascular endothelial growth factor and basic fibroblast growth factor in the induction of angiogenesis *in vitro*. *Biochem. Biophys. Res. Commun.*, **189**, 824–831.

PORSCHEN R, PORSCHEN W, MÜHLENSIEPEN H AND FEINENDEGEN LE. (1983). Cell loss from viable and necrotic tumour regions measured by $^{125}$I-UdR. *Cell Tissue Kinet.*, **16**, 549–556.

RICE GC, SPIRO IJ AND LING CC. (1985). Detection of S-phase overreplication following chronic hypoxia using a monoclonal anti-Brd Urd. *Int. J. Radiat. Oncol. Biol. Phys.*, **11**, 1817–1822.

SHWEIKI D, ITIN A, SOFFER D AND KESHET E. (1992). Vascular endothelial growth factor induced by hypoxia may mediate hypoxia-initiated angiogenesis. *Nature*, **359**, 843–845.

SIEMANN DW, LORD EM, KENG PC AND WHEELER KT. (1981). Cell subpopulations dispersed from solid tumours and separated by centrifugal elutriation. *Br. J. Cancer*, **44**, 100–108.

SOUTTER AD, NGUYEN M, WATANABE H AND FOLKMAN J. (1993). Basic fibroblast growth factor in urine of bladder cancer patients. *Cancer Res.* **53**, 5297–5299.

VLODAVSKY I, FUKS Z, ISHAI-MICHAELI R, BASHINK P, LEVI E, KORNER G, BAR-SHAVIT R AND KLAGSBRUN M. (1991). Extracellular matrix-resident basic fibroblast growth factor: implication for the control of angiogenesis. *J. Cell. Biochem.* **45**, 167–176.

WEST CML, KENG PC, SIEMANN DW AND SUTHERLAND RM. (1987). A human colon adenocarcinoma xenograft – radiation response, cellular composition, and tumor disaggregation. *J. Natl Cancer Inst.*, **78**, 371–376.